# A spiking network model for passage-of-time representation in the cerebellum

**DOI:** 10.1111/j.1460-9568.2007.05837.x

**Published:** 2007-10

**Authors:** Tadashi Yamazaki, Shigeru Tanaka

**Affiliations:** 1 Laboratory for Visual Neurocomputing, RIKEN Brain Science Institute 2–1 Hirosawa, Wako, Saitama 351–0198, Japan; 2 Department of Brain Science and Engineering, Graduate School of Life Science and Systems Engineering, Kyushu Institute of technology 2–4 Hibikino, Wakamatsu-ku, Kitakyushu-shi, Fukuoka, 808–0196, Japan

**Keywords:** cerebellum, eyeblink conditioning, NMDA, passage of time, recurrent network

## Abstract

In Pavlovian delay eyeblink conditioning, the cerebellum represents the passage-of-time (POT) between onsets of conditioned and unconditioned stimuli (CS and US, respectively). To study possible computational mechanisms of the POT representation we built a large-scale spiking network model of the cerebellum. Consistent with our previous rate-coding model, we found two conditions necessary for the present model to represent the POT with a dynamic population of active granule cells: (i) long temporal integration of input signals; and (ii) random recurrent connections between granule and Golgi cells. When these conditions were satisfied, a nonrecurrent sequence of active granule cell populations was generated in response to a CS and, conversely, the POT from the CS onset was able to be read out from the sequence. Specifically, simulated *N*-methyl-*D*-aspartate (NMDA) channels with a long decay time constant at granule and Golgi cells were responsible for the long temporal integration. Thus, blocking the NMDA channels or ablating Golgi cells impaired the POT representation. Simulated glomerulus structure made POT representation robust against noise in mossy fibre inputs. Long-term potentiation induced at mossy fibre synapses on granule cells also served to enhance the robustness. We reproduced some experimental results of Pavlovian delay eyeblink conditioning using the present model. These results suggest that the recurrent network in the granular layer and NMDA channels in granule and Golgi cells play an essential role in the timing mechanisms in the cerebellum, whereas the glomerulus serves to realize a robust representation of time.

## Introduction

The cerebellum plays an essential role in motor learning and control. One function of the cerebellum is to represent the passage of time (POT) over a range of tens to hundreds of milliseconds, a function essential for organising movements of different body parts into a coordinated action (Ivry & Spencer, 2004). The POT representation in the cerebellum has been studied in depth using Pavlovian delay eyeblink conditioning (see Mauk & Donegan, 1997 and Christian & Thompson, 2003, for review), in which an animal receives repeated paired presentations of a tone (conditioned stimulus; CS) and an airpuff (unconditioned stimulus; US). The animal becomes conditioned to close its eyes with a delay equal to the interstimulus interval (ISI) between the CS and US onsets (conditioned response; CR) in response to the tone. Thus, the animal learns the POT between the CS and US onsets. How then is the POT represented in the cerebellum?

One hypothetical way to represent the POT is to assign one neuron or one neuron population to one time interval from the CS onset. If active neurons or neuron populations are sequentially generated in the order of interval lengths and one neuron or population exclusively corresponds to only one time interval, then we regard the sequence of these neurons or populations as the POT from the CS onset. Based on this hypothesis, several artificial models have been proposed to account for the POT representation (Fujita, 1982; Moore *et al*., 1989; Gluck *et al*., 1990; Chapeau-Blondeau & Chauvet, 1991; Bullock *et al*., 1994; Fiala *et al*., 1996). Buonomano & Mauk (1994) and Medina *et al*. (2000) questioned the biological plausibility of these models and built a realistic cerebellar model. They demonstrated that a POT representation based on a time-varying population of granule cells emerged naturally from the realistic model. The computational mechanism to generate such a sequence of active populations of granule cells, however, remained unclear. To understand the computational mechanism, we previously developed a rate-coding model of the cerebellar granular layer and analysed its dynamics theoretically (Yamazaki & Tanaka, 2005a). We have shown that when the CS is given, granule cells exhibit random repetition of transitions between active and inactive states and that different granule cells show different transition patterns. The activity pattern of granule cells evolved with time, and the sequence of active granule cell populations did not recur for a sufficiently long time. This indicates that there is a one-to-one correspondence between an active granule cell population and a time step. Two conditions were needed for the generation of a nonrecurrent sequence of active granule cell populations. One is a temporal integration of input signals over long time, which enabled active or inactive states of individual granule cells to be sustained. The other is random recurrent connections, which enabled granule cells to undergo random transitions between active and inactive states. The rate-coding model is free from actual time scales. Construction of an elaborated model is therefore desired for quantitative comparison with a biological system.

In the present study, we built a large-scale spiking network model of the cerebellum and examined how the POT is represented in the elaborated model. We also studied the robustness of the POT representation against noise in mossy fibre (MF) inputs. Finally, we conducted simulation of Pavlovian delay eyeblink conditioning to test whether the model reproduced experimental results. Preliminary results have been reported in an abstract (Yamazaki & Tanaka, 2005b).

## Materials and Methods

### Overview of the present model

#### Network structure

**Fig. 1:**
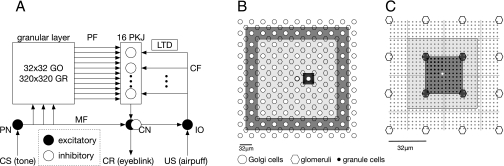
(A) Schematic of cell types and synaptic connections incorporated into the present model with the flow diagram of neural signals of the CS, US and CR.(B) Spatial arrangement of model Golgi cells (circles), glomeruli (hexagons) and granule cells (dots). Shaded rectangles in pale and dark grey represent, respectively, the dendritic and the axonal arborization of the model Golgi cell at the centre.(C) Spatial arrangement of model glomeruli (hexagons) and granule cells (dots) in detail. The rectangle in pale grey represents the dendritic arborization of the model granule cell at the near centre (white dot). For example, 100 granule cells in the grey box (black dots) contact the four glomeruli surrounding these granule cells (filled hexagons).

Figure 1A is a schematic of cell types and synaptic connections incorporated in the present model with the flow diagram of neural signals of the CS, US and CR. The neural signal of the CS is conveyed through MFs, from the precerebellar nucleus (PN) to the cerebellar nucleus (CN). The CN neuron fires spikes representing the motor command for an eyeblink. On the other hand, the CS signal is also sent to Purkinje cells relayed by the granule cells in the granular layer through parallel fibres (PFs), and then Purkinje cells inhibit the CN. In this way, the CN receives direct excitatory inputs and indirect inhibitory inputs from the PN. The granular layer contains Golgi cells as well as granule cells. Golgi cells receive excitatory inputs from granule cells and recurrently inhibit granule cells. The neural signal of the US comes from the inferior olive (IO) through the climbing fibre (CF) to Purkinje cells. The CN neuron inhibits the IO. The CR is represented by the spikes fired by the CN neuron after eyeblink conditioning.

Figure 1B is a schematic of the model granular layer composed of model Golgi cells, glomeruli and granule cells. Our artificial cerebellum modelled as a 1 mm^2^ virtual sheet composed of a square lattice arrangement of 1024 (32 × 32) model Golgi cells with a constant spacing of 32 µm. This virtual sheet also contains 320 × 320 model granule cells and 32 × 32 model glomeruli, all of which are arranged in a square lattice, so the nearest neighbour spacing of glomeruli is the same as that of Golgi cells.

Figure 1C illustrates connections between model granule cells and glomeruli. A model granule cell has four dendrites and receives excitatory inputs from MFs and inhibitory inputs from model Golgi cells via glomeruli. We assumed that a model granule cell contacts the four nearest glomeruli. This leads to a nested structure of the granular layer due to the square arrangement of granule cells: 10 × 10 granule cells contact commonly the same glomeruli, which we call ‘granule-cell clusters’. Thus, 100 granule cells in a cluster share the same excitatory and inhibitory inputs.

A glomerulus is assumed to receive inhibitory inputs from 9 × 9 nearby model Golgi cells with a connection probability of *P* = 0.025, so that the average number of Golgi cell axons innervating a glomerulus is ∼2.0 (9 × 9 × 0.025). Hence, a granule cell receives, on average, 8.1 inhibitory inputs through four dendrites. A glomerulus also receives an excitatory MF input, so that granule cells contacting the same glomerulus should receive a common excitatory signal. However, to simulate the stochastic variability of synaptic transmission from a glomerulus to granule cells, we modelled individual granule cells contacting a glomerulus to receive different Poisson spikes with the same firing rate (see ‘Stimulus’ section for details).

A model Golgi cell is assumed to be able to receive inputs from 700 × 700 model granule cells, namely 7 × 7 granule cell clusters. Connections from a granule-cell cluster to a Golgi cell are made with probability *P* = 0.5, where individual granule cells belonging to a common granule-cell cluster have the same connection. We also examined two other configurations: (i) a Golgi cell receives inputs from 7 × 7 granule-cell clusters with probability *P* = 1.0; (ii) a Golgi cell receives inputs from only 10 × 10 granule cells in the nearest cluster with *P* = 1.0. In both cases, the POT was still represented (data not shown).

The above-mentioned structural parameters of our model are selected on the basis of the following anatomical observations. The Golgi cell density in the cat granular layer is ∼1000/mm^3^ (Palkovits *et al*., 1971b; Lange, 1974). We projected this volume into a 1-mm^2^ virtual sheet which contained almost the same number of model Golgi cells. In the same volume, there are 1000× more granule cells than Golgi cells in cats (Palkovits *et al*., 1971b). Because it is impossible to perform a computer simulation with 1 million model neurons due to the power of our computers, in the present model we reduced the number of granule cells to 320 × 320, which is ∼1/10 of the actual number of granule cells. The number of glomeruli was also reduced so that the average distance between nearest neighbour glomeruli was 32 µm; the distance in cats is reported to be 18.4 µm (Palkovits *et al*., 1972). We assumed that individual granule cells contacted the four nearest glomeruli. This seems reasonable when we consider that in cats the average length of granule cell dendrites is 13.59 µm (Palkovits *et al*., 1972), which is shorter than 18.4 µm of the average distance between neighbouring glomeruli (Palkovits *et al*., 1972). It has been reported that the size of maximal axonal arborization of a Golgi cell is 300 × 300µm^2^ (Dieudonné, 1998). This range of the arborization covers 9 × 9 model Golgi cells, where the model Golgi cell spacing was assumed to be 32 µm. This justifies a glomerulus receiving inhibitory inputs from 9 × 9 nearby model Golgi cells. When we determined that connections from granule cells to Golgi cells ranged over 7 × 7 granule-cell clusters, we relied on the following experimental findings: (i) the average size of the dendritic arborization of a Golgi cell is 200 × 200µm^2^ (Dieudonné, 1998), and (ii) Golgi cell inhibition produced by a parallel fibre volley extends transversely no farther than 200 µm (Eccles *et al*., 1967). The area 200 × 200µm^2^ contains 7 × 7 granule-cell clusters in our model.

In cats, 330 Purkinje cells are contained in 1 mm^2^ of tissue of the cerebellar cortex (Palkovits *et al*., 1971a). We assumed that in the model cerebellar cortex of 1 mm^2^, 16 model Purkinje cells were aligned sagittally with a cell spacing of 64 µm. The average size of the dendritic arborization of Purkinje cells is 292 µm (Palkovits *et al*., 1971a), whereas PFs elongate 1 mm in the present model. Thus, a model Purkinje cell receives inputs from model granule cells within 292 × 1000 µm^2^. Because the distance between two nearby granule-cell clusters was set at 32 µm, the model Purkinje cell receives inputs from 9 × 32 granule-cell clusters (292 µm ∼9 × 32 µm). Finally, each granule-cell cluster contains 10 × 10 granule cells, so the Purkinje cell receives inputs from 90 × 320 granule cells. All model Purkinje cells also received CF inputs from the model IO. We considered only one neuron in the model CN as an output neuron of the system; it received excitatory MF inputs and inhibitory inputs from all model Purkinje cells. This model CN neuron was assumed to inhibit the IO neuron effectively. This simplification was justified by the presence of inhibitory neurons in the CN sending axons to the IO neurons (De Zeeuw & Berrebi, 1995). We also assumed one neuron in the model IO which transmits the US signal.

Thus, we built a minimal model of the cerebellar cortex which was able to represent and learn the POT. Therefore, we omitted all the other types of neurons such as basket, stellate, Lugaro and unipolar brush cells. We also omitted trigeminal inputs to the CN given directly and indirectly via the IO. We confirmed that, in the simulation of eyeblink conditioning, the peristimulus time histogram (PSTH) of the CN neuron in the model with the IO input did not differ from that in the model without the IO input except that within 50 ms immediately after the US onset the firing rate of the CN neuron transiently increased.

#### Neuron models

Neurons were modelled as conductance-based, leaky integrate-and-fire units. (1)
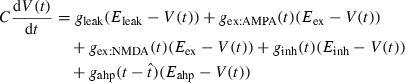
 where *V*(*t*) and *C* are the membrane potential at time *t* and the capacitance, respectively, and 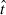
is the last firing time of the neuron. The membrane potential was determined by five types of currents specified by the right-hand side of equation 1, namely, leak, α-amino-3-hydroxy-5-methyl-4-isoxazolepropionic acid receptor (AMPAR)-mediated, *N*-methyl-*D*-aspartate receptor (NMDAR)-mediated and γ-aminobutyric acid type A receptor (GABA_A_R)-mediated currents, and the current for emulation of the after-hyperpolarization. For each type *C* (where subscript *c*∈{leak, ex:AMPA, ex:NMDA, inh, ahp}), the current was calculated from the conductance *g*_*c*_ and reversal potential *E*_*c*_. A conductance was calculated from the convolution of the alpha function α(*t*) and the spike event dgr _*j*_(*t*) of presynaptic neuron *j* at time *t* as follows: (2)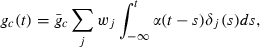 where 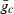
represents the maximum conductance and *w*_*j*_ the synaptic weight from the presynaptic neuron *j*. The alpha functions were defined for each current and each neuron type with different time constants. When the membrane potential of a neuron exceeded the threshold θ the neuron was deemed to fire a spike, followed by the after-hyperpolarization that determined the refractory period. The conductance for the after-hyperpolarization was given by (3)
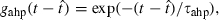
where tgr _ahp_ represents the time constant of the after-hyperpolarization and 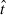
is the last firing time of the neuron (Gerstner & Kistler, 2002). Parameter values were adopted from known physiological data as shown in Tables 1 and 2. Neurons of the same type were assumed to be identical: cell parameters were the same among these neurons. We found that the POT is represented robustly because the network dynamics was not sensitive to the choice of cell parameters within a physiologically appropriate range. Thus, exact values of parameters and their variability among individual neurons were not important in the present model.

**Table 1:** Summary of cell parameters

	Neuron type				
	
Cell parameters	GR	GO	PKJ	CN	IO
θ (mV)	−35.0	−52.0	−55.0	−38.8	−50.0
*C* (pF)	3.1	28.0	107.0	122.3	10.0
*g*_leak_ (nS)	0.43	2.3	2.32	1.63	0.67
*E*_leak_ (mV)	−58.0	−55.0	−68.0	−56.0	−60.0
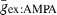 (nS)	0.18	45.5	0.7	50.0	1.0
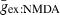 (nS)	0.025	30.0	–	25.8	–
*E*_ex_ (mV)	0	0	0	0	0
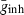 (nS)	0.028	–	–	30.0	0.18
*E*_inh_ (mV)	−82.0	–	–	−88.0	−75.0
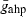 (nS)	1.0	20.0	0.1	50.0	1.0
*E*_ahp_ (mV)	−82.0	−72.7	−70.0	−70.0	−75.0
τ _ahp_ (ms)	5.0	5.0	5.0	2.5	10.0

GR, granule cell (D'Angelo *et al*., 1995; Brickley *et al*., 1999); GO, Golgi cell (Midtgaard, 1992; Maex and De Schutter, 1998; Dieudonné, 1998); PKJ, Purkinje cell (Puia *et al*., 1994; De Schutter and Bower, 1994); CN, cerebellar nucleus neuron (Czubayko *et al*., 2001; Mouginot and Gähwiler, 1995); IO, inferior olivary neuron (Bal and McCormick, 1997; Schweighofer *et al*., 1999; Llinás and Yarom, 1981); –, nonexistent.

**Table 2:** Summary of alpha functions

Neuron type	Alpha function
GR	α _ex:AMPA_ (*t*) = e^–*t*/1.2^
	α _ex:NMDA_ (*t*) = e^–*t*/52.0^
	α _inh_ (*t*) = 0.43e^–*t*/7.0^ + 0.57e^–*t*/59.0^
GO	α _ex:AMPA_ (*t*) = e^–*t*/1.5^
	α _ex:NMDA_ (*t*) = 0.33e^–*t*/31.0^ + 0.67e^–*t*/170.0^
PKJ	α _ex:AMPA_ (*t*) = e^–*t*/8.3^
CN	α _ex:AMPA_ (*t*) = e^–*t*/9.9^
	α _ex:NMDA_ (*t*) = e^–*t*/30.6^
	α _inh_ (*t*) = e^–*t*/42.3^
IO	α _ex:AMPA_ (*t*) = e^–*t*/10.0^
	α _inh_ (*t*) = e^–*t*/10.0^

GR, granule cell (Silver *et al*., 1992; Puia *et al*., 1994; D'Angelo *et al*., 1995; Brickley *et al*., 1999); GO, Golgi cell (Dieudonné, 1998); PKJ, Purkinje cell (Llano *et al*., 1991); CN, cerebellar nucleus neuron (Audinat *et al*., 1992; Mouginot & Gähwiler, 1995); IO, inferior olivary neuron, defined arbitrarily.

#### Synaptic weights

Granule cells fire 25 spikes/s when two dendrites are repetitively stimulated at 50 Hz under *E*_leak_ = −60 mV (D'Angelo *et al*., 1995). Two simultaneous spikes on different dendrites are necessary for a granule cell to produce a spike (Gabbiani *et al*., 1994). Golgi cells in the cat cerebellum fire up to 100 spikes/s while the cat is walking (Edgley & Lidierth, 1987). During the eyeblink conditioning experiments, Purkinje cells fire up to 100 spikes/s when the CS is presented (Kotani *et al*., 2003). Neurons in the CN also produce up to, on average, 100 spikes/s when the CS is presented (Delgado-Garc'a & Gruart, 2002). We determined synaptic weights so that the firing rates of model neurons were similar to these experimental data.

#### Stimulus

**Fig. 2:**
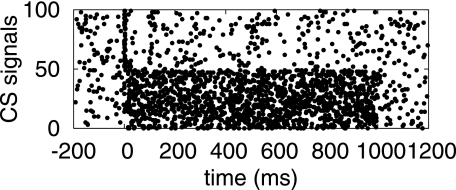
Spike patterns of the sustained and transient components of MF input signals. The first 50 examples are of sustained components whereas the rest are of transient components. When *t* < 0, both components were set to be 5-Hz Poisson spikes. The CS onset was set at *t* = 0. The sustained component was 30-Hz Poisson spikes for 1 s. The transient component consisted of 200-Hz Poisson spikes for the first 5 ms followed by 5-Hz Poisson spikes. In the 5-ms interval immediately after the CS onset, on average one spike was elicited.

A model granule cell receives four excitatory inputs from four nearby glomeruli, so that 100 model granule cells in a cluster receive the same excitatory inputs. Because cell parameters and synaptic weights are identical at individual cells, these granule cell must produce identical responses under this setting. On the other hand, real granule cells exhibit a variety of response patterns across cells and trials because of individual variability of cell parameters and synaptic weights, stochasticity in channel dynamics and action potential generation, and so on. For example, the decay time constant of excitatory postsynaptic potentials (EPSPs) of granule cells is known to be 74.0 ± 33.8 ms (D'Angelo *et al*., 1995), indicating that the deviation is almost 50% of the mean value. Such variability and stochasticity jitter the onset of EPSPs and spike timing at individual granule cells, which could be effectively simulated by random shift of the spike timing of MFs. Thus, although model granule cells in a cluster contact the same four glomeruli, excitatory signal fed into individual granule cells via glomeruli were assumed to be independent for each cell. Eventually, we attempted to model MF signals as four independent Poisson spikes fed into each granule cell without any other variability. The spontaneous activity level of the pontine nuclei of adult rats is ∼5 Hz (Freeman & Muckler, 2003). The neural signal of the CS consists of transient and sustained components (Aitkin & Boyd, 1978; Freeman & Muckler, 2003). The ratio of the transient to the sustained units is almost 1 : 1 (Freeman & Muckler, 2003). The firing rate of the sustained component is ∼30 Hz (Freeman & Muckler, 2003). According to these experimental findings, first we fed 5-Hz Poisson spikes into MFs for 1 s prior to the CS presentation in order to set the network activity to a steady state. Then, at *t* = 0, we fed the CS signal consisting of transient and sustained components. The sustained component was modelled as 30-Hz Poisson spikes for 1 s. The transient component was modelled as 200-Hz Poisson spikes for the first 5 ms followed by 5-Hz Poisson spikes. Thus, in the first 5 ms, on average one spike was elicited. Each granule cell received two transient and two sustained components of the CS signals through four dendrites. Figure 2 illustrates firing patterns of sustained and transient components. The first 50 firing patterns are of sustained components whereas the rest are of transient components, where the CS onset was set at *t* = 0. The model CN neuron, on the other hand, received one transient and one sustained components of the CS signals. The US signal, which was a transient current strong enough to elicit a spike, was given to the IO neuron. The CS signal was sustained for 1 s even after the US arrival in order to confirm that the present model elicited the CR only around the US presentation. This differs from delay eyeblink conditioning experiments, in which the CS coterminates with the US.

#### Synaptic plasticity

We simulated long-term depression (LTD) and long-term potentiation (LTP) at PF synapses on Purkinje cells according to experimental findings (LTD: Ito, 1989, 2001, 2002; LTP: Lev-Ram *et al*., 2003; Coesmans *et al*., 2004). For simulated LTP, the synaptic weight of PF *j* at Purkinje cell *i* denoted by *w*_PKJ*i*←PF*j*_ was updated at each time *t* as follows: (4)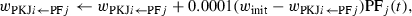 where PF_*j*_(*t*) = 1 if PF *j* fires a spike at *t*, and 0 otherwise, and *w*_init_ denotes the initial PF synaptic weight set at 1. The simulated LTP amplifies the active PF synaptic weight by 0.0001. For simulated LTD, *w*_PKJ*i*←PF*j*_ was updated at the end of the CS as follows: (5)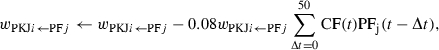 where CF(*t*) = 1 if the IO fires a spike at time *t* and 0 otherwise. This simulated LTD reduces the synaptic weight of PF *j* that is active at 0–50 ms before the CF input by a factor of 0.08.

Plasticity at MF synapses on the CN neuron has also been reported (Racine *et al*., 1986; Caria *et al*., 2001). This plasticity seems to be related to the representation of the amplitude of the CR, not the timing of the CR. Kleim *et al*. (2002) have reported that synapse formation on CN neurons is associated with memory storage. Boyden *et al*. (2004) have suggested that the CN is more important for storing information about the response strength whereas the cerebellar cortex stores timing-related information. Shutoh *et al*. (2006) have shown that memory of the learned response is first formed in the cerebellar cortex and then only memory of the response amplitude is transferred to and consolidated at the CN. Because we focused on how the timing of the CR is learned rather than the consolidation of the CR amplitude, we fixed synaptic weights of MF inputs to the model CN neuron.

#### Simulation tools

The simulation program was written in C programming language. Differential equations were numerically solved using the 4th order Runge–Kutta method with a fixed step size of 1 ms.

### Data analysis

Let *z*_*i*_(*t*) be the population average activity of a granule-cell cluster *i*, defined as the total amount of AMPAR-mediated EPSPs to Purkinje cells divided by the number of granule cells in a cluster, as follows: (6)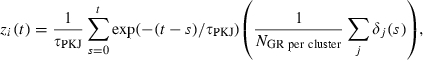 where dgr _*j*_(*s*) represents the spike elicited in model granule cell *j* in the cluster, *N*_GR per cluster_ is the number of granule cells in a cluster (namely, 100) and τ_PKJ_ is the decay time constant of AMPAR-mediated EPSPs at Purkinje cells, which was set at 8.3 ms.

We studied how the activity pattern of granule cell clusters evolved over time. To do this, first we defined the autocorrelation of the activity pattern at times *t* and *t* + Δ*t* as follows: (7)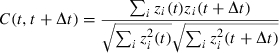 The numerator represents the inner product of activity pattern vectors at time *t* and *t* + Δ*t*, and the denominator normalizes the vector lengths. Because *z*_*i*_(*t*) takes only positive values the correlation takes a value between 0 and 1. It would be 1 if the activity pattern vectors at time *t* and *t* + Δ*t* are identical, and it would be 0 if the vectors are orthogonal, indicating that the activity patterns have no overlap.

We then defined the similarity index *S*(Δ*t*) as follows: (8)
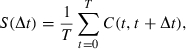
where *T* represents the duration of the CS signal (namely, 1 s). This is the average of equation 7 with respect to *t*. This index represents how two activity patterns separated by Δ*t* are correlated, on average. If the similarity index decreased as Δ*t* increased, it indicates that an activity pattern evolved with time into uncorrelated patterns. We defined the standard deviation of the similarity index as well: (9)
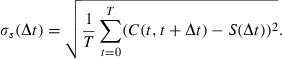
 We also defined the reproducibility index *R*(*t*) as follows: (10)
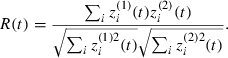

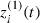
and 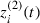
are activity patterns of granule-cell cluster *i* at time *t* for two different input signals (e.g. input signals with different noise). The reproducibility index represents how two activity patterns for different input signals differ from each other as time elapses.

### Simulation of delay eyeblink conditioning

In the simulation of delay eyeblink conditioning, we tested three ISIs: 250, 500 and 750 ms. For each ISI, paired presentation of the CS and the US was given 100 times and the PSTH of the model CN neuron was calculated.

In experiments, a PSTH is calculated using data obtained from CS-alone trials to avoid contamination from the strong influence of US signals. On the other hand, in this study, neither complex spike generation at Purkinje cells induced by CF signals nor excitatory connections from the IO to the CN were modelled. Moreover, update of PF synaptic weights by simulated LTD was made at the end of each trial. Taken together, the US input via CF had no influence on the network dynamics. Therefore, we did not distinguish CS-alone trials from CS–US trials and used spike data of the CN neuron during CS–US trials to obtain the PSTH.

## Results

### POT representation by sparse granule-cell populations

The simulated network was given 5-Hz Poisson spikes that represent average spontaneous activity of neurons in the precerebellar nucleus (Freeman & Muckler, 2003) for 1 s prior to the presentation of the CS, which led to steady-state activity of the network. In this steady state, granule and Golgi cells fired spikes randomly. The average firing rate of granule cells was ∼5 Hz. In contrast, Maex & De Schutter (1998) have reported that granule and Golgi cells fire spikes synchronously at 20 Hz in the steady state in their granular layer model. The synchronized activity, however, was not retained at 5 Hz. We examined whether granule and Golgi cells in the present model undergo synchronization at a higher frequency by increasing the firing rate of Poisson spikes fed into the network. However, we did not observe such synchronization.

**Fig. 3:**
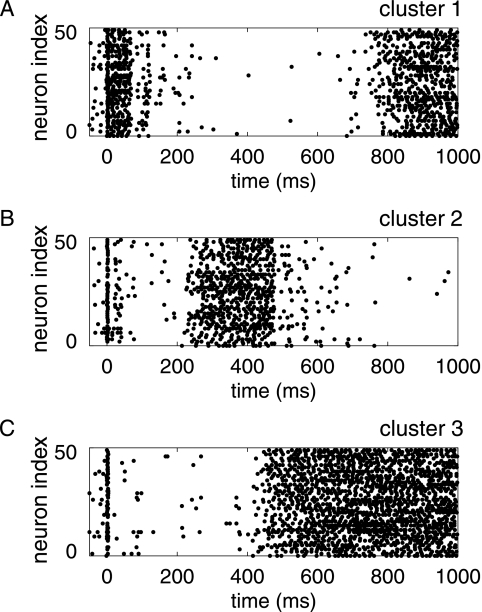
(A) Spike patterns of 50 out of 100 granule cells chosen randomly in a granule-cell cluster. Abscissa and ordinate represent time and neuron index, respectively. The CS onset was set at *t* = 0. Because they received the same inhibitory inputs they exhibited similar spike trains, although individual spike timing varied from cell to cell. This indicates that these granule cells behaved as a functional unit as a whole. (B and C) Spike patterns of 50 granule cells in two other clusters. The granule cells in each cluster exhibited similar spike patterns whereas spike patterns were quite different among different clusters.

The simulated CS was presented to the network at *t* = 0. Figure 3A shows the spike patterns of 50 out of 100 granule cells in a cluster. These granule cells contact the same MF terminals and share common inhibitory inputs from the same Golgi cells. In spite of the stochastic spike trains conveyed by MFs, the granule cells exhibited similar spike patterns, in which they tended to fire spikes from 0 to 100 ms and from 800 to 1000 ms. Figure 3B and C shows the spike patterns of 50 granule cells in two different clusters. Granule cells in cluster 2 fired spikes frequently from 250 to 500 ms, whereas granule cells in cluster 3 quite probably generated spikes from 500 to 1000 ms. Again, the spike patterns were similar across granule cells in the same cluster. Such a similarity of spike activities of granule cells suggests that a granule-cell cluster behaves as a functional unit that represents information robustly against stochastic fluctuation inherent in the CS. This result also demonstrates that granule cells belonging to different clusters exhibited different temporal activity patterns.

**Fig. 4:**
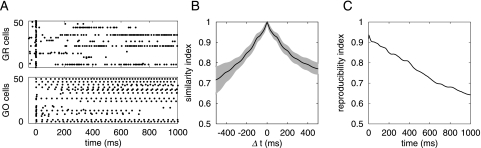
(A) Spike patterns of 50 granule cells (GR; top) and Golgi cells (GO; bottom). Granule cells undergwent random repetition of transitions between burst and silent states. Different granule cells exhibited different temporal spike patterns. Golgi cells fired spikes rather regularly. All conventions are as in Fig. 3.(B) The similarity index *S*(Δ*t*) defined in equation 8 is plotted with a solid line and the standard deviation sgr _s_(Δ*t*) defined in equation 9 is represented by the grey region. The similarity index monotonically decreased from 1 at Δ*t* = 0 as Δ*t* increased. This indicates that the population of active granule cells changed gradually with time, and no population appeared more than once for 1 s.(C) The reproducibility index defined by equation 10. Abscissa and ordinate represent the time from the CS onset and the index, respectively. The reproducibility was highest at the beginning and decreased gradually with time, retaining a high value, suggesting that two sequences of active granule cell populations generated by two different Poisson spike trains were almost identical at the beginning of the stimulation, and then they gradually became dissimilar with time, retaining a high reproducibility. The minimum similarity and reproducibility indices were 0.72 and 0.64, respectively.

The top panel in Fig. 4A shows the spike patterns of 50 granule cells that were randomly chosen from each granule-cell cluster. As mentioned above, these granule cells chosen from different clusters revealed different temporal activity patterns. Specifically, they underwent random repetition of transitions between burst and silent states. The burst state was sustained for tens to hundreds of milliseconds. On the other hand, Golgi cells fired spikes rather regularly as shown by the bottom panel. Figure 4B shows the similarity index of the activity pattern against the time shift Δ*t*. This figure indicates that the population of active granule cells changed gradually with time and that no active granule-cell populations appeared more than once. These properties guarantee the one-to-one correspondence between the active granule-cell population and the POT from the CS onset. The matrix describing random inhibitory connections from Golgi to granule cells maps a population of active granule cells into another, dissimilar, population of active granule cells. This computation is a kind of ‘random projection’. This random projection was repeated during the CS presentation and the random sequence of active granule cell populations was generated. The simulation demonstrated that, on average, only 0.65% of granule cells (∼ 650 cells) fired spikes for the time interval of 1 ms. In other words, the POT from the CS onset can be represented by a gradually changing active granule-cell population in a scheme of sparse coding.

**Fig. 5:**
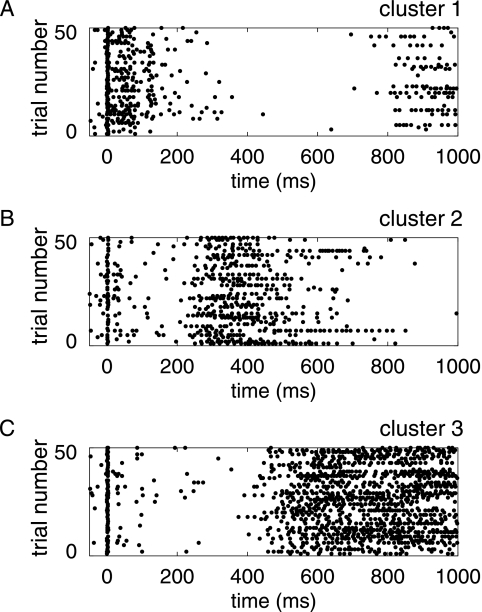
Spike patterns of a granule cell chosen randomly in the same three clusters as Fig. 3 for 50 trials. Ordinate represents the trial number; the other conventions are as in Fig. 3. Across trials, these three granule cells exhibited similar temporal spike patterns. Variability of spike patterns across trials seems similar to that of spike patterns across cells shown in Fig. 3.

Information representation in a sparse coding scheme seems vulnerable to the noise inherent in the external input signal. In our network, how robustly can temporally changing active granule-cell populations represent POT? We conducted two simulations using different Poisson spike trains fed into MFs, comparing the granule cell activity patterns obtained from the two simulations. As shown in Fig. 4C, two activity patterns generated by different Poisson spike trains with the same mean firing rate were almost identical at the beginning of the stimulation but gradually became dissimilar, retaining a high reproducibility. As shown in Fig. 5, we confirmed this property by plotting spike patterns of identical granule cells for 50 different Poisson spike trains with the same mean firing rate, where three granule cells were taken from the three clusters that are shown in Fig. 3. In cluster 1 (Fig. 5A), the granule cell tended to fire spikes at high frequency from 0 to 100 ms across different trials of stimulation, whereas the cell probably fired spikes sparsely after 800 ms for one-third of the trials. In clusters 2 and 3 (Fig. 5B and C), similar spike patterns were observed across different trials and the patterns became more variable with time. The high reproducibility indicates that the random structure of alternating transitions between burst and silent states at granule cells was not due to the temporal jitter of Poisson spikes fed into MFs. More importantly, the spike patterns of a single granule cell in a cluster across different trials shown in Fig. 5 were quite similar to those of different granule cells in the same cluster in a single trial shown in Fig. 3. This observation implies that the trial average activity of a single granule cell in a cluster is almost identical to the population average activity of many granule cells in the same cluster in a single trial. A Purkinje cell receives signals from many PFs and hence its membrane potential is computed by the population average activity of granule cells. Based on this property of spatial integration and the equivalence between the population average and the trial average, it is thought that a Purkinje cell computes the trial average activity of granule cells. Thus, Purkinje cells represent timing information robustly in spite of the variability of signals in the CS across different trials.

### Effects of blocking NMDA channels on POT representation

As shown in Fig. 4A, the burst periods of granule cells sustained for several tens to hundreds of milliseconds. This raises the question of whether particular synaptic channels are involved in determining the duration of a burst state and a silent state. Experiments using granule cell slices indicate that temporal summation of input signals is mainly determined by the NMDAR-mediated EPSPs; there is a larger decay time constant of NMDAR-mediated EPSPs (52.0 ms) than that of AMPAR-mediated EPSPs (1.2 ms; Silver *et al*., 1992). It is quite likely that simulated NMDAR-mediated current maintained the burst states. To test this possibility, we carried out simulation of the network dynamics blocking NMDA channels, in which the conductance of NMDA channels was set to 0 at either granule cells or Golgi cells.

**Fig. 6:**
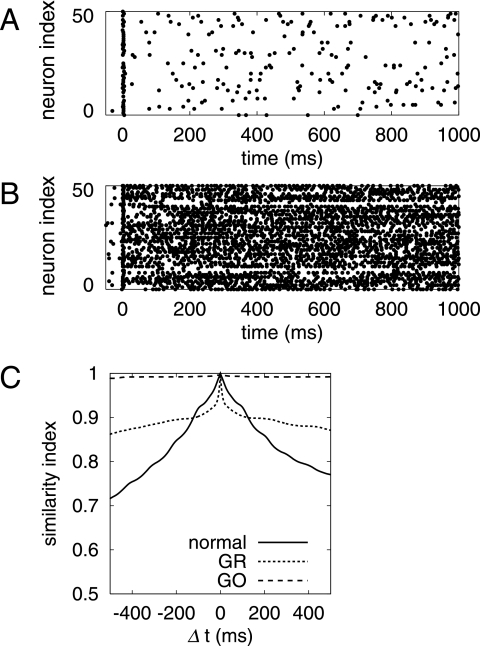
(A) Spike patterns of 50 granule cells when NMDA channels of granule cells were blocked. Each neuron was chosen randomly from 50 different granule-cell clusters. Because of the failure of temporal integration of MF signals at granule cells over a long time in the absence of NMDAR-mediated current they fired spikes sparsely and did not exhibit burst- and silent-state transitions.(B) Spike patterns of 50 granule cells when NMDA channels at Golgi cells were blocked. Golgi cells decreased their firing rate due to the absence of NMDAR-mediated current. In turn, reduced inhibition made granule cells fire spikes vigorously. Again, they did not exhibit burst- and silent-state transitions. All conventions are as in Fig. 3.(C) Similarity indices when NMDA channels of granule cells were blocked (GR, dotted line) and when those of Golgi cells were blocked (GO, dashed line). In both cases, the plot of similarity indices is flat except the obvious similarity around *t* ≈ 0, indicating that there was no temporal structure in granule cells' activity patterns. The solid line is the similarity index under the normal condition, which is the same as in Fig. 4.

Figure 6A shows the spike patterns of 50 granule cells under the blockade of NMDA channels at granule cells. The maximum firing rate decreased from 79 to 17 spikes/s. This drastic reduction in firing rate was attributed to the failure of temporal integration of MF signals over a long time at granule cells in the absence of NMDAR-mediated current. Figure 6B represents the spike patterns under the blockade of NMDA channels at Golgi cells. The maximum firing rate of Golgi cells decreased from 47 to 16 spikes/s due to the absence of NMDAR-mediated current to Golgi cells. On the other hand, the maximum firing rate of granule cells reached as high as 90 spikes/s. The enhanced firing rate of granule cells was attributed to the reduction in inhibition from the less active Golgi cells. As expected, in both cases granule cells no longer showed clear alternating transitions between burst states and silent states, but rather fired spikes at almost constant frequencies. As there was no temporal structure in the activity patterns, similarity indices for both cases became flat, as shown in Fig. 6C. This indicates the disruption of the POT representation. Consequently, temporal integration of input signals based on long-lasting depolarization induced by NMDAR-mediated current generated a random sequence of transitions between burst states and silent states.

### Enhancement of robustness in POT representation

**Fig. 7:**
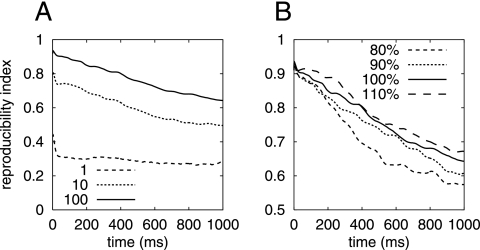
(A) Reproducibility indices for different numbers of granule cells in a cluster: 1, 10 and 100. A larger number of granule cells per cluster enhanced reproducibility of the sequence generation of active granule cell populations.(B) Reproducibility indices for different weights of MF synapses on granule cells: 80, 90, 100 and 110%. Larger synaptic weights enhanced the reproducibility. All conventions are as in Fig. 4C.

As shown in Fig. 4C, the POT representation was robust against temporal jitter of MF spikes. We questioned which mechanisms support this robustness. First, we examined whether the huge number of granule cells in the cerebellum makes the POT representation robust. We conducted simulation with 100, 10 or 1 granule cells per cluster. Figure 7A shows the plot of the reproducibility index for each case. The lower bounds of values of the reproducibility index were 0.64, 0.50 and 0.28 for 100, 10 and 1 granule cells per cluster, respectively. As the number of granule cells decreased, the reproducibility became worse. This suggests that, in the cerebellum, redundancy in information representation of input signals due to the huge number of granule cells serves to achieve the robust representation of POT. Because the number of granule cells in the cerebellum is 10× larger than that assumed in the present model (Palkovits *et al*., 1971b; Ito, 1984), the POT representation in the real cerebellum should be more robust than that in the present model.

Second, we examined the effect of the MF synaptic weight at granule cells on the robustness of the POT representation. We carried out simulations for different strengths of the synaptic weight. As shown in Fig. 7B, the reproducibility increased as the synaptic weight increased. The lower bounds of values of the reproducibility index were 0.57, 0.61, 0.64 and 0.67 for −20, −10, 0 and 10% changes in the synaptic weight, respectively. Taken together with the findings of LTP induced at MF synapses on granule cells (D'Angelo *et al*., 1999; Armano *et al*., 2000; Hansel *et al*., 2001), this LTP may contribute to enhancement of the robustness in the POT representation.

### Representation of multiple time passages

How does the model work when different patterns of input signals are fed into MFs? We conducted simulations in which only half of the granule-cell clusters that were randomly chosen were stimulated by the CS signal while the rest were exposed to the 5-Hz background activity. Then we reversed the set of CS-stimulated granule cells and the set of granule cells exposed to the background activity and conducted the simulation again. The maximum value of the reproducibility index between the two stimulation conditions was found to be <0.1. This low reproducibility suggests that different activity patterns were generated when MFs were stimulated with different patterns of CS signals. We also confirmed that the activity pattern of granule cells under each simulation condition can represent a POT specified by the input signal. These results suggest that the representation of multiple POTs can be embedded into the same granule–Golgi cell circuit, and the information on individual POTs can be read out separately for different CS signals.

### Learned timing by Purkinje cells

Next, we conducted simulations of the network to show how model Purkinje cells learn and represent the ISI between the CS onset and the timing of US presentation. In the simulation, we repeatedly presented the same CS–US pair 100 times. While a spatiotemporal activity pattern of granule cells generated by the CS signal input was sent to Purkinje cells through PFs, the US signal was fed into the IO 500 ms after the onset of the CS. In order to confirm that the present model elicited the CR only around the US presentation, we continued the CS presentation for 1 s even after the US presentation, although the CS typically coterminates with the end of the US in standard eyeblink conditioning protocols.

**Fig. 8:**
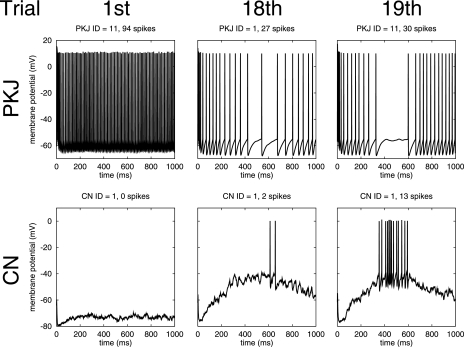
Membrane potentials of a Purkinje cell (top panels) and the CN neuron (bottom panels) at the first, 18th and 19th trials (from left to right). Spikes are marked by vertical lines. Abscissa and ordinate in each panel represent the time from the CS onset and the membrane potential, respectively. PKJ, Purkinje cell. Because the US was given at 500 ms, Purkinje cells learned to stop firing at ∼500 ms as conditioning trials were repeated. On the other hand, the CN neuron disinhibited by Purkinje cells began to fire spikes at ∼500 ms. This indicates the generation of the CR. Note that the first spike of the CN neuron was elicited slightly earlier than the US onset.

The top panels in Fig. 8 show membrane potentials of a Purkinje cell at the first, 18th and 19th trials of the conditioning. At the first trial, the cell exhibited a constant high-frequency spike train at 94 spikes/s. As conditioning was repeated, the firing rate decreased, particularly around the time of the US presentation. As shown above in *POT representation by sparse granule-cell populations*, a nonrecurrent sequence of active granule cell populations was generated during the CS presentation and the sequence generation was reproducible across trials. Thus, the population of active granule cells at the time of the US presentation was uniquely determined across trials, and only their PF synapses were depressed. Therefore, the net input to the Purkinje cell decreased around the US presentation. On the other hand, long before and after the US presentation the net input did not change because LTD was not induced at synapses due to the lack of coincidence between the CS and the US. The temporally localized reduction in the net input to a Purkinje cell around the US presentation decreased the firing rate of the Purkinje cell. Such a decrease in the Purkinje cell firing rate has been found experimentally (Berthier & Moore, 1986; Hesslow & Ivarsson, 1994; Kotani *et al*., 2003; Jirenhed *et al*., 2007). Moreover, the simulation showed that the overall firing rate of the Purkinje cell decreased trial-by-trial. This decrease was due to the fact that some granule cells that were active when the US was presented could be active again at the other times and the LTD of synapses of these granule cells contributed to the reduction in the net input to the Purkinje cell. The tendency toward reduction in the firing rate over the course of conditioning has been observed in a recent experiment (Kotani *et al*., 2006).

The bottom panels in Fig. 8 show membrane potentials of the CN neuron in the first, 18th and 19th conditioning trials. In the first trial, the neuron was not able to fire spikes because it was strongly inhibited by Purkinje cells. In the 18th trial, the CN neuron fired a few spikes because Purkinje cell inhibition became weaker around the US presentation. It should be noted, however, that the first spike was elicited after the US presentation. At the 19th trial the neuron fired more spikes, representing a vigorous CR. Moreover, the first spike appeared at ∼380 ms, which is earlier than the US presentation. This may represent the so-called anticipatory CR (Ohyama *et al*., 2003).

After the 19th trial, the CN neuron inhibited the IO neuron earlier than the US presentation due to the anticipatory spike output of the CN neuron. Therefore, the IO neuron failed to fire spikes necessary for the induction of LTD at Purkinje cells. This suppressed further induction of LTD, which may serve to avoid over-learning.

### Simulation of delay eyeblink conditioning

**Fig. 9:**
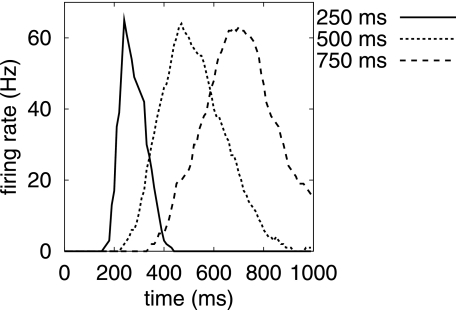
PSTH of the CN neuron for 100 trials of the conditioning simulation. The ISIs were set at 250, 500 and 750 ms. The firing rate reached a maximum at the same time as the ISI. As the ISI increased, the maximum firing rate slightly decreased while the width of the histogram increased. Abscissa and ordinate represent the time from the CS onset and the firing rate of the CN neuron, respectively.

In the simulation of delay eyeblink conditioning, the ISI was set at 250, 500 or 750 ms and, for each ISI, paired presentation of the CS and the US was given 100 times. The PSTH of the model CN neuron was plotted in Fig. 9. For any ISI, the CN neuron did not produce spikes for a while after the CS onset because the neuron was strongly inhibited by Purkinje cells. After the silent period of the CN neuron, the firing rate steadily increased with time and reached a maximum at the same time as the ISI. This is consistent with experimental findings (McCormick & Thompson, 1984a; McCormick & Thompson, 1984b; Berthier & Moore, 1990). After that, the firing rate sharply decreased in spite of sustained presentation of the CS. Moreover, the maximal firing rate slightly decreased and the width of the histogram increased as the ISI increased. Such ISI-dependent behaviour seems to correlate with experimental results in which the amplitude of eyelid closure, as measured by electromyography (EMG), decreased while the eyelid response trace became broader as the ISI increased (Mauk & Ruiz, 1992).

### Effects of Golgi cell ablation on eyeblink conditioning

Watanabe *et al*. (1998) have studied the role of Golgi cells in motor coordination in mice by selectively ablating Golgi cells using an immunotoxin-mediated cell targeting technique. They found that the Golgi cell ablation caused severe acute motor disorders. They also found that elimination of Golgi cells not only reduced GABAR-mediated inhibition but also attenuated the NMDAR-mediated current to granule cells. The attenuation of NMDAR-mediated current may compensate for the reduction in inhibition from Golgi cells, by which the balance between the excitation and inhibition acting on granule cells may be achieved. This possibility is partially supported by the tendency of Golgi cell-ablated mice to show gradual recovery (Hirano *et al*., 2002). We tested whether the present model can reproduce the above findings.

**Fig. 10:**
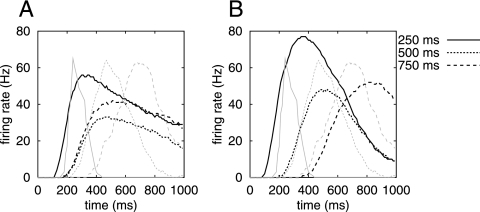
PSTH of the CN neuron for 100 trials of the conditioning simulation under(A) Golgi cell ablation and under(B) Golgi cell ablation and reduction in the NMDA channel conductance on granule cells. The ISIs were set at 250, 500 and 750 ms. Thin lines show PSTH in the normal case. Ablating Golgi cells caused the time of peak firing rate to shifts, and the firing rate did not return to 0 after the US presentation. Successive reduction in the NMDA channel conductance of granule cells rescued the timed peak of the firing rate although slightly shifted, whereas the sustained firing after the US presentation was still preserved. All conventions are as in Fig. 9.

We carried out simulation of eyeblink conditioning, randomly eliminating 80% of Golgi cells in the network. The PSTH illustrated in Fig. 10A shows that the time of peak firing rate was advanced (for ISI 750 ms) or delayed (for ISI 250 ms), whereas the maximal firing rate decreased by 10% (ISI 250 ms) or >30% (ISIs of 500 and 750 ms). Moreover, the neuron tended to continue producing spikes long after the US presentation. The similarity index in this case became almost flat and its minimum value was 0.95. These results imply a failure of the POT representation as well as a failure of conditioning.

Next, we conducted simulation of eyeblink conditioning, reducing the peak conductance of NMDA channels on granule cells by 40% in addition to the 80% elimination of Golgi cells. The simulated PSTH in Fig. 10B shows that the maximal firing rate increased and the temporal localization of spike activity was restored again. In particular, the maximal firing rate for an ISI of 250 ms exceeded that in the normal case. On the other hand, the time of peak firing rate was delayed when the ISI was set at either 250 or 750 ms. Moreover, the width of the PSTH was still broader than that in the normal conditioning case. This suggests that the suppression of NMDAR-mediated current into granule cells tends to restore the learning capability that has been lost by the ablation of Golgi cells. However, the broadening of the PSTH implies that the performance of CRs is still worse than that in the normal conditioning case. Thus, the experimental finding of gradual recovery of CRs was successfully reproduced by the simulations of our network model under the elimination of Golgi cells combined with the reduction in the peak conductance of NMDA channels.

### Effect of blocking NMDA channels on eyeblink conditioning

**Fig. 11:**
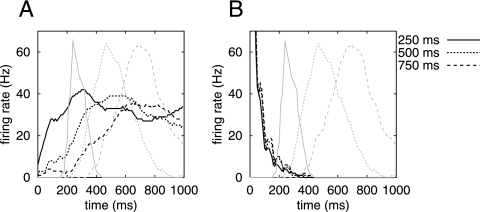
PSTH of the CN neuron for 100 trials of the conditioning simulation under the blockade of NMDA channels on(A) granule cells and(B) Golgi cells. The ISI was set at 250, 500 and 750 ms. Thin lines show PSTH in the normal case. Blocking NMDA channels on granule cells caused the CN neuron to start to fire spikes earlier than in the normal case, and it did not stop firing after the US presentation. Hence, the peak firing rate at the corresponding ISI is not observed clearly. On the other hand, blocking NMDA channels on Golgi cells abolished the timed peak of the firing rate completely. All conventions are as in Fig. 9.

Finally, we examined how the blockade of NMDA channels on granule or Golgi cells affected the conditioning. Figure 11A shows the PSTH of the CN neuron for 100 trials when the peak conductance of NMDA channels on granule cells was set at 0. The PSTH peak appeared at the corresponding ISIs. However, the CN neuron started to produce spikes earlier and did not stop firing after the US presentation. Moreover, the overall firing rate significantly decreased. Thus, the PSTH became almost flat around and after the US presentation. This property suggests uncertainty in the CR even after the conditioning under the blockade of NMDA channels. NMDAR subunits consist of two classes: NR1 and NR2 (NR2A–NR2D). Granule cells express both NR2A and NR2C; in particular, the mRNA for NR2C is expressed only in granule cells in the cerebellum (Watanabe *et al*., 1994). NMDAR-mediated EPSPs in granule cells characterized by a long time constant are nearly abolished in knockout mice lacking both NR2A and NR2C, and these mice fail to stay on a rotating rod, indicating severe motor dyscoordination (Kadotani *et al*., 1996). These results suggest that NMDAR-mediated EPSPs are indispensable for precise motor control. We found that 51 trials were necessary for the CN neuron to start eliciting a vigorous CR that completely suppressed spike generation by the IO neuron, whereas 19 trials were sufficient in the normal case. These simulation results predict that learning in animals without the function of NMDARs on granule cells proceeds much more slowly than that in normal animals. This prediction is consistent with the experiment by Kishimoto *et al*. (1997), demonstrating that mutant mice lacking both NR2A and NR2C showed slower acquisition of the CR by 400 trials of the conditioning, whereas 140 trials were sufficient in wild-type mice.

On the other hand, the blockade of NMDA channels on Golgi cells completely impaired the conditioning, as shown in Fig. 11B. The CN neuron fired spikes at high frequencies immediately after the CS was given, and then the firing rate monotonically decreased with time. We examined the firing pattern of Purkinje cells and found that they fired isolated spikes synchronously over all Purkinje cells shortly after the CS onset. The CN neuron could produce spikes during the intervals between the successive Purkinje spikes due to the release of the CN neuron from the Purkinje cells' synchronized inhibition. This induced high-frequency spike activity immediately after the CS onset. Spikes fired by the Purkinje cells, however, became gradually desynchronized with time. This desynchronization led to continuous inhibition of the CN neuron by Purkinje cells. Therefore, the PSTH of the CN neuron decreased as time elapsed.

## Discussion

### POT representation in the model cerebellar granular layer

Accumulating evidence seems to support the hypothesis that the cerebellar cortex represents the POT (Perrett *et al*., 1993; Garcia & Mauk, 1998; Ohyama & Mauk, 2001; Bao *et al*., 2002), but the mechanism is still a matter of debate. Several models have been proposed (Fujita, 1982; Moore *et al*., 1989; Gluck *et al*., 1990; Chapeau-Blondeau & Chauvet, 1991; Bullock *et al*., 1994; Buonomano & Mauk, 1994; Fiala *et al*., 1996; Medina *et al*., 2000). Among them, representation of the POT using a recurrent network composed of granule and Golgi cells was first studied by Buonomano & Mauk (1994). The model was later elaborated more realistically by the same group (Medina *et al*., 2000) and their simulation reproduced some features of CRs in eyeblink conditioning experiments. However, the mechanism by which a population of active granule cells could represent the POT was not clear. Focusing on that mechanism, we studied the dynamics of a simplified rate-coding model of a granule and Golgi cell circuit (Yamazaki & Tanaka, 2005a) and found two necessary conditions for the POT representation: long temporal integration of input signals and random connections between granule and Golgi cells. The simplified model is advantageous in elucidating principal mechanisms qualitatively, owing to its mathematical tractability. On the other hand, it is unlikely to be useful for quantitative argument in comparison with a real system. In the present study, we built a spiking network model of the cerebellar circuit using integrate-and-fire units as model neurons, and examined to what extent the computational mechanism found in the simplified model is valid for a spiking network model. We found several important properties of the network underlying the POT representation in the cerebellum.

First, time-varying inhibition from Golgi cells to granule cells through random recurrent connections shaped a spatiotemporal structure in the activity patterns of granule cells. Granule cells exhibited random repetition of transitions between burst and silent states, and different granule cells showed different firing patterns. Thus, no population of active granule cells appeared more than once. Because the active granule cell population changed with time, the spatiotemporal pattern represented the POT. Several lines of evidence suggest that the recurrent inhibition shapes the spatiotemporal neuronal activity patterns in the brain. For example, in the olfactory bulb, mitral cells receive odour signals and excite granule cells, which in turn inhibit mitral cells. Mitral cells exhibit complex spatiotemporal activity patterns when odours are presented (Laurent *et al*., 2001), and the spatiotemporal patterns are shaped by inhibition (Wilson & Laurent, 2005). In the prefrontal cortex, excitatory neurons show a variety of temporal discharge patterns triggered by a sensory cue stimulus during a delay interval in delayed-response tasks (Niki & Watanabe, 1979; Kojima & Goldman-Rakic, 1982; Compte *et al*., 2003). The temporally irregular persistent activity is caused by inhibition (Constantinidis *et al*., 2002).

Second, NMDAR-mediated EPSPs that have a large decay time constant made granule and Golgi cells integrate input signals over a long time, and so their spiking periods were sustained up to hundreds of milliseconds. Because of the slow dynamics, the active granule cell population changed gradually over time and represented the POT. Blocking of simulated NMDA channels on granule and Golgi cells disabled the sustained spike generation and hence the POT representation was disrupted. This result also explains why the model of Medina *et al*. (2000) represents the POT: they assumed a long decay time constant of PSPs (75 ms from MFs to granule cells and 50 ms from Golgi cells to granule cells). Experimental evidence of the involvement of NMDAR-mediated EPSPs in delayed and/or sustained responses has been reported. For example, Imamura *et al*. (2000) have found that electrical stimulation to the white matter of cerebellar slices induces postsynaptic long-lasting depolarization in the granular layer, but the depolarization disappeared when an NMDA antagonist 2-amino-5-phosphonovalerate (APV) was applied. Buonomano (2003) has demonstrated the induction of reliably timed action potentials with a delay as long as 300 ms after a single-pulse stimulation to cortical slices. The delayed responses were caused by the retention of activity by the network dynamics. The activity retention was found to be mediated mainly by NMDAR activation because the delayed responses disappeared when APV was applied to the slices. These studies suggest that the large decay time constant of NMDAR-mediated EPSPs is necessary for the recurrent circuit to retain the activity.

Third, the simulated glomerulus provided 100 nearby granule cells with common inhibitory inputs. These granule cells exhibited similar firing patterns in spite of noisy MF signals due to jittering of the spike timing. Thus, they represented MF signals redundantly and assisted in transmitting signals to Purkinje cells robustly. Firing patterns of a single granule cell in a cluster across different trials (Fig. 5) were similar to those across different granule cells in the same cluster in a single trial (Fig. 3). This suggested that the averaged activity of a single granule cell across different trials, which is constant across trials, could be approximated by calculating the averaged activity of many PFs at Purkinje cells. LTP at MF synapses on granule cells increased the average firing rate of granule cells and thereby decreased the variance of the interspike interval across different trials. The smaller variance indicates less fluctuation of EPSPs at Purkinje cells. Thus, these operations enhanced the reproducibility of the POT representation across different trials.

Finally, oscillation in field potentials has been reported in the granular layer of immobile animals (Edgley & Lidierth, 1987; Pellerin & Lamarre, 1997; Hartmann & Bower, 1998). Maex & De Schutter (1998) have built a model of the cerebellar granular layer using the Hodgkin–Huxley neuron model and observed synchronization of granule and Golgi cell firing. Their model did not represent the POT because of the absence of NMDA channels at Golgi cells whereas ours did not exhibit synchronization; this may be due to the absence of Hodgkin–Huxley-type channel dynamics in our model neurons. In a related study, we attempted to switch the network dynamics between synchronization and POT representation by changing the strength of MF signals (Yamazaki & Tanaka, 2006).

The present model lacks several details, such as MF input to Golgi cells, voltage-dependency of NMDA receptors, tonic inhibition in glomeruli, unipolar brush cells and so on. In this respect, the present model is still far from the real cerebellum. We would like to emphasize that our model is devoted to providing a minimal cerebellar model for the POT representation.

### Simulation of eyeblink conditioning

Our model successfully reproduced some experimental observations in Pavlovian delay eyeblink conditioning. Purkinje cells learned to stop firing around the time of US presentation through LTD at PF synapses, and the CN neuron was thereby released from inhibition to elicit the CR. Once robust CR was established, the CN neuron inhibited the IO. This inhibition to the IO suppressed over-learning, resulted in a longer duration of the silent period of Purkinje cells and was beneficial for retaining the representation of precise timing. At the same time, LTP overtook LTD; this cancelled out accidentally occurring LTD and stabilized the PF synaptic weights (Kenyon *et al*., 1998).

In Fig. 9, the width of the PSTH is seen to increase as the ISI increased; this emerged from the ISI-dependent reduction in reproducibility of granule cell activity patterns (Fig. 4C). Chen & Thompson (1995) found that the induction level of LTD at PF synapses on Purkinje cells depends on the time interval between PF and CF stimulation. Kotaleski *et al*. (2002) have demonstrated that the elevation in intracellular Ca^2+^ concentration is also ISI-dependent, using a detailed model Purkinje cell. Thus, multiple mechanisms may participate in the ISI dependency.

### Computational power of cerebellar cortex

In the Marr–Albus–Ito theory of cerebellar computation (Marr, 1969; Albus, 1971; Ito, 1984), the cerebellum is considered to be a perceptron (Rosenblatt, 1958) in which granule and Purkinje cells constitute the input and output layers, respectively, and PF synapses are able to learn under the instruction of CF signals. The spatial pattern of MF input signals is encoded by a combination of active granule cells; this is called codon representation (Marr, 1969) or expansion recoding (Albus, 1971). In our model, as described in Results in *Representation of multiple time passages*, a combination of active granule-cell clusters, not individual granule cells, represents the spatial pattern of MF signals. Moreover, the POT from the stimulus onset is also represented in the same combinatorial coding. In this sense, we naturally extend the conventional idea of combinatorial representation in the granular layer to the spatiotemporal domain.

On the other hand, the cerebellum may not be a biological counterpart of a perceptron because the computational power of a perceptron is so limited while the cerebellum must have universal computational capability to form internal models (Wolpert *et al*., 1998). Recently, a liquid state machine (LSM) has been proposed as a new framework for neural computation (Maass *et al*., 2002). A recurrent circuit of neurons generates spatiotemporal activity patterns called liquid states; readout neurons receive the liquid states and external instruction signals, and learn to extract time-varying information. For computational power, an LSM is better than a perceptron. We have noticed that the structure of the present model fits an LSM (Yamazaki & Tanaka, 2007): the spatiotemporal activity pattern of model granule cells, model Purkinje cells and CF signals can be regarded as cerebellar counterparts of liquid states, readout neurons and instruction signals to readouts. The contemporary view of the cerebellar circuitry may be more analogous to an LSM than a perceptron. Our model provides a theoretical tool for exploring the computational power of the cerebellum from the viewpoint of the LSM.

## Abbreviations

AMPAR: α-amino-3-hydroxy-5-methyl-4-isoxazolepropionic acid receptor
CF: climbing fibre
CN: cerebellar nucleus
CR: conditioned response
CS: conditioned stimulus
EPSP: excitatory postsynaptic potential
GABA_A_R: γ-aminobutyric acid type A receptor
IO: inferior olive
ISI: interstimulus interval
LTD: long-term depression
LTP: long-term potentiation
MF: mossy fibre
NMDA: *N*-methyl-*D*-aspartate
OS: conditioned stimulus
PF: parallel fibre
PN: precerebellar nucleus
POT: passage of time
PSTH: peristimulus time histogram
US: unconditioned stimulus.
